# The Alteration Mechanism of Copper-bearing Biotite and Leachable Property of Copper-bearing Minerals in Mulyashy Copper Mine, Zambia

**DOI:** 10.1038/s41598-019-50519-z

**Published:** 2019-10-21

**Authors:** Gai-rong Wang, Hong-ying Yang, Yuan-yuan Liu, Lin-lin Tong, Ali Auwalu

**Affiliations:** 1 School of Metallurgy, Northeastern University, Shenyang, 110819 China; 2 CNMC Luanshya Copper Mines Plc (CLM). Independence Avenue, Luanshya City, 90456 Zambia

**Keywords:** Geochemistry, Mineralogy

## Abstract

The XRF, XRD, polarizing microscopy and SEM-EDS were used to study the alteration mechanism of copper-bearing biotite and the leachable property of copper-bearing minerals in Mulyashy Copper Mine, Zambia. It was found that biotite can be divided into copper-bearing biotite and copper-free biotite. Some copper-bearing biotite existed in the form of monomer, and others aggregated with copper-bearing chlorite, malachite or copper-bearing limonite. The main reason for the occurrence of biotite aggregations was that copper-bearing biotite underwent two kinds of alteration mechanisms as follows: altering into copper-bearing chlorite and malachite, and altering into copper-bearing chlorite and copper-bearing limonite. The order of factors effecting the copper leaching rate of the ores in acid leaching experiments was temperature > sample size > H_2_SO_4_ concentration > leaching time > stirring speed. In addition, the copper leaching rate of copper-bearing minerals at different temperatures was in the following order: malachite, chrysocolla and pseudomalachite > copper-bearing chlorite > copper-bearing muscovite > copper-bearing biotite > copper-bearing limonite. The leachable property of biotite is closely related to its special structure.

## Introduction

The copper deposits in the world are widely distributed across five continents including more than 150 countries^1^. According to the proportion of oxide minerals and sulfide minerals in copper ores, it can be divided into sulfide ores containing copper oxide less than 10%, mixed ores containing copper oxide of 10–30% and oxide ores containing more than 30% copper oxide^2^. With the increasing depletion of sulfide ores and high grade copper oxide ores, there is a need to win metals from the abundant complex copper oxide ores^3–5^. The ores have the characteristics of low copper grade, high oxidation rate, high combined rate, fine distribution granularity as well as extremely complex copper phase composition^6,7^. The process mineralogy of the ores from Mulyashy Copper Mine in Luanshya, Zambia showed that it was a kind of typical complex copper oxide ores and the copper minerals mainly contained malachite, chrysocolla, pseudo-malachite, limonite, biotite, muscovite, chlorite, in which biotite was one of the significant copper-bearing minerals^8^.

Biotite is an important ferric silicate mineral with the molecular formula of (K, Na, Ca, Ba) (Fe^2+^, Fe^3+^, Mg, Ti^4+^, Mn, Al)_3_(Al, Si)_4_O_10_(OH, F, Cl)_2_ ^9^. Most previous works on chemical composition of biotite from porphyry Cu deposits paid attention to determining the contents of F and Cl, with the aim of identifying mineralized and barren plutons^10–12^. Due to the appearance of Fe^2+^, Fe^3+^ and OH^−^, it was reported that biotite could also be used to evaluate the physicochemical conditions in the crystallization process of some minerals as well as the thermodynamic parameters like T(°C), f(O_2_) and f(H_2_O)^13^. In addition, many scholars focused on the exchanged improved coefficient of F-Cl-OH between biotite and hydrothermal fluid, calculating the values of some parameters such as log (fH_2_O/fHCl), log (fHCl/fHF), log (X_Cl_/X_OH_), log (X_F_/X_OH_) and log (X_F_ /X_Cl_), which can indicate the process of hydrothermal alteration and mineralization^14–16^. However, because of their own special characteristics of layered structures, biotite minerals often appear as the carriers or enrichment minerals of many metallogenic elements, such as Cu, Au, *etc*^17^.

The content and occurrence of copper in biotite minerals have been studied by some scholars since 1963. It was reported that the copper content in biotite was about 6 × 10^−6^–5000 × 10^−6^, which was related to whether it was metallogenic intrusion or not^18,19^. Earlier studies indicated that the copper might not exist in the form of sulfide^20^, but in the form of octahedral coordination in biotite^21^. However, it was recently considered that the abnormal copper existed in the form of natural copper inclusions and expansive intercalations in biotite, and the inclusions and expansive intercalations were only found in the biotite with epigenetic weathering^22–24^. It was further indicated that the biotite with epigenetic weathering had the phenomenon of higher copper and lower potassium^25^. Liu *et al*. discovered that biotite mineralization associated with Cu was rich in magnesium and poor in iron^17^. Moreover, it has also been found that the copper occurred in biotite of goethite belt existed in two forms: natural copper inclusions and iron oxides rich in copper, and the content of CuO in iron oxides was as high as 5%, indicating that the iron was mostly replaced by copper^26^. However, recently seldom studies have been concentrated on the alteration mechanism of copper-bearing biotite and leachable property of copper-bearing minerals in copper ores.

For the reasons above, the study was carried out to analyze the mineral characteristics of copper-bearing biotite by means of advanced detection methods such as X-ray fluorescence (XRF), X-ray diffractomer (XRD), polarizing microscopy and scanning electron microscope equipped with an energy dispersive spectrometer (SEM-EDS), which can further provide theoretical value and significant guidance for the leaching of copper contained in biotite minerals.

## Results and Discussion

It was found from previous stable isotopic work that many porphyry copper deposits were related to the early stage alteration (e.g. potassic) caused by fluids of magmatic origin, and the later stage alteration (e.g. phyllic) superimposed on earlier alteration zones, caused principally by meteoric fluids^27,28^. In addition, the K-feldspar-quartz and chlorite-sericite-pyrite alteration associations are more important in the formation of porphyry copper deposits^29^. However, it was shown from Kahang porphyry copper deposit that biotite could alter into chlorite and opaque minerals such as goethite and magnetite^30^. In general, the biotite alteration is slightly earlier than the main mineralization, while Banks suggested that the enrichment of copper in intrusive rocks near Arizona porphyry copper deposit occurred during the process of biotite altering into chlorite^31^. In this paper, it was discovered that there were two existence forms of biotite, copper-bearing biotite and copper-free biotite, respectively. And the copper-bearing biotite could differentiate and alter into other minerals along with its dissociation and cleavage crack unlike the copper-free biotite.

### The occurrence of copper in copper-bearing biotite

Some scholars inferred that the abnormal copper in the altered biotite did not occupy the position of lattice octahedron, but existed in the form of natural copper inclusions and expansive intercalations in interlayer domain of biotite. However, it was shown from the SEM-EDS analysis of this study that the copper in altered biotite mainly existed in the state of isomorphism by replacing magnesium or iron, and this copper-bearing biotite continued to differentiate and alter into copper-bearing chlorite and other copper oxide minerals.

**Figure 1:**
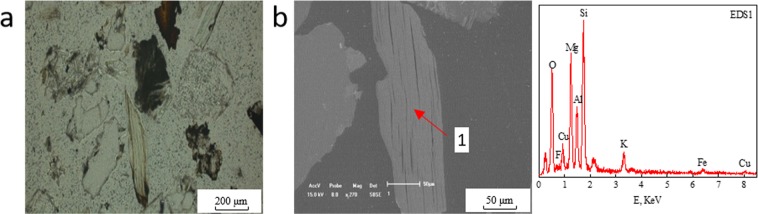
Planar polarizing microscopy and SEM-EDS diagram of monomeric copper-bearing biotite. (**a**) Planar polarizing microscopy. (**b**) SEM; EDS1. The spectra corresponds to point 1 in SEM diagram.

**Figure 2:**
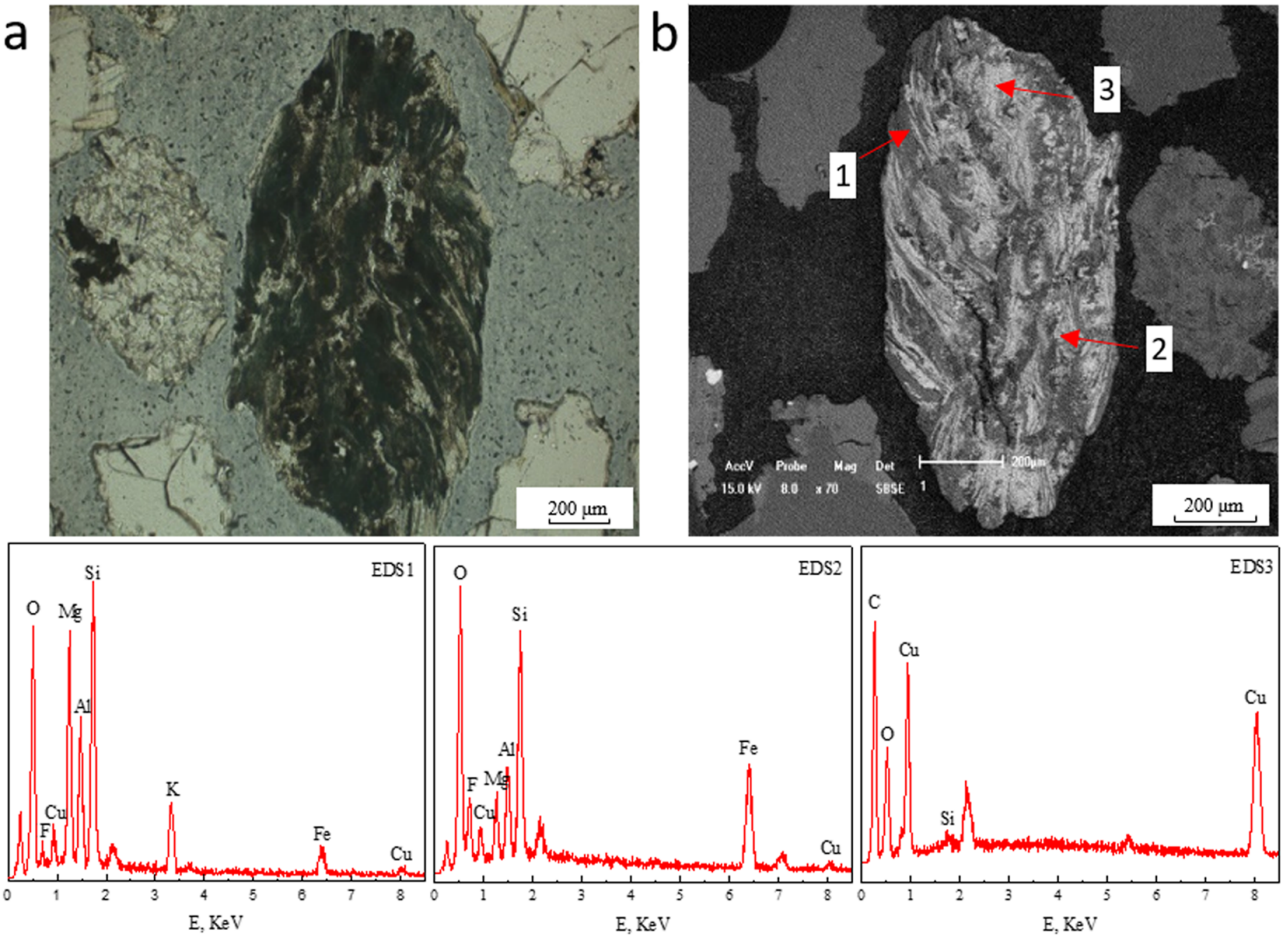
Planar polarizing microscopy and SEM-EDS diagrams of copper-bearing biotite altering into copper-bearing chlorite and malachite. (**a**) Planar polarizing microscopy. (**b**) SEM; EDS1, EDS2, EDS3. The spectra correspond to points 1, 2, 3 in SEM diagrams.

**Figure 3:**
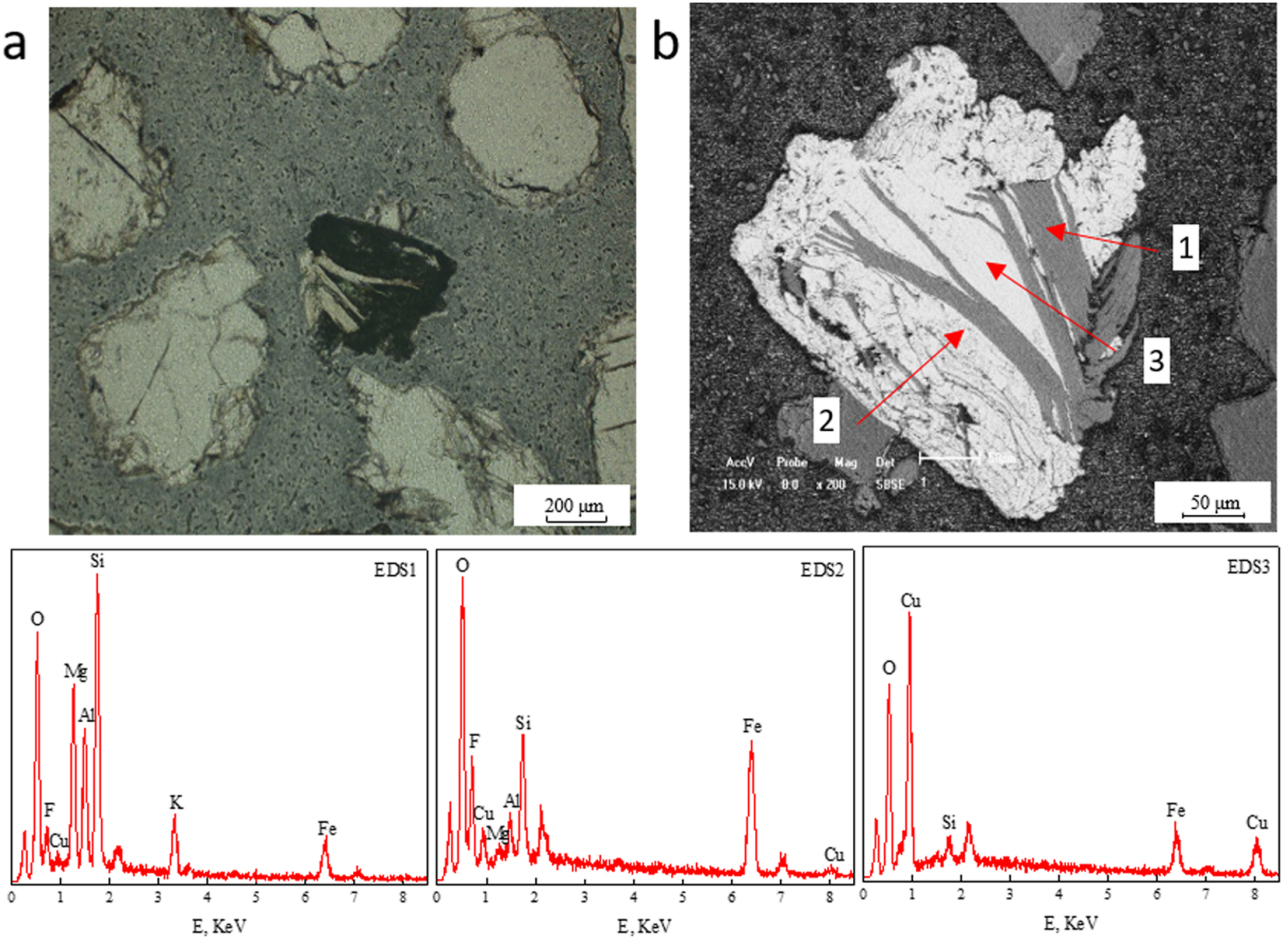
Planar polarizing microscopy and SEM-EDS diagrams of copper-bearing biotite altering into copper-bearing chlorite and copper-bearing limonite. (**a**) Planar polarizing microscopy. (**b**) SEM; EDS1, EDS2, EDS3. The spectra correspond to points 1, 2, 3 in SEM diagrams.

It was detected that there were two forms of copper-bearing biotite: monomeric copper-bearing biotite and biotite aggregations in which the copper-bearing biotite occurred with other copper minerals such as copper-bearing chlorite, malachite or copper-bearing limonite. Figure 1 shows the planar polarizing microscopy and SEM-EDS diagrams of monomeric copper-bearing biotite. Figures 2 and 3 present the planar polarizing microscopy and SEM-EDS diagrams of biotite aggregations with copper minerals. In addition, the energy spectrum analysis (EDS) of the two forms of biotite are presented in Tables 1 and 2 ^32^, which indicated that the Cu contents were significantly different between the two kinds of biotite. It was found that the copper content in monomeric biotite was 1.23–6.01% with average value of 2.83%, which was obviously lower than that of 2.23–7.38% in biotite aggregations with the average content of 4.27%. In addition, from the SEM-EDS analysis of monomeric copper-bearing biotite and biotite aggregations, it was observed that the color varied from light gray to deep gray depending on the amount of copper. The biotite with higher copper showing light-gray color, usually is lighter than the biotite with lower copper or copper free biotite which shows dark-gray color^32^. However, the color of malachite is usually white due to its highest copper content.

**Table 1:** Energy spectrum analysis data of monomeric biotite (Wt%).

No.	O	F	Mg	Al	Si	K	Fe	Co	Cu	Total
1	44.65	1.62	12.46	9.44	19.26	7.30	2.88	0	2.39	100
2	38.31	1.43	9.90	11.08	22.00	8.45	7.32	0.17	1.34	100
3	47.15	0.83	8.17	10.86	18.85	5.65	6.20	0.23	2.06	100
4	48.86	4.85	6.51	10.59	16.75	4.36	4.54	1.01	2.53	100
5	50.81	3.02	11.73	7.44	16.01	2.07	1.61	1.51	5.80	100
6	39.13	3.31	10.02	10.00	20.38	6.31	8.60	1.02	1.23	100
7	48.62	1.17	11.98	7.84	15.57	4.23	3.27	1.31	6.01	100
8	45.08	2.02	13.95	9.86	18.56	4.85	3.32	0.98	1.38	100
9	43.29	0.98	14.73	9.27	18.71	4.39	3.34	2.01	3.28	100
10	46.76	2.97	13.46	8.97	17.93	2.53	4.04	1.03	2.31	100
Avg.	45.27	2.22	11.29	9.54	18.40	5.01	4.51	0.93	2.83	100

**Table 2:** Energy spectrum analysis data of biotite aggregations (Wt%).

No.	O	F	Mg	Al	Si	K	Fe	Co	Cu	Total
1	49.26	4.63	8.37	9.56	19.40	2.45	1.29	0.22	4.82	100
2	47.71	5.75	11.09	6.72	17.22	1.08	1.77	1.28	7.38	100
3	53.15	2.02	9.48	10.73	17.33	0.88	1.74	0.44	4.23	100
4	49.91	2.99	7.41	11.60	19.53	1.42	3.61	0.43	3.10	100
5	56.19	2.68	8.98	8.29	16.92	1.37	2.32	1.02	2.23	100
6	54.49	2.01	8.78	9.86	15.16	3.15	2.23	0.47	3.85	100
7	52.58	3.23	12.28	7.67	15.21	3.29	0.82	0.72	4.20	100
8	57.03	2.61	6.59	12.08	11.76	1.89	1.87	0.61	5.56	100
9	53.41	3.42	11.74	7.43	16.01	2.07	1.61	1.50	2.81	100
10	56.04	1.30	12.95	8.04	12.40	3.22	1.14	0.40	4.51	100
Avg.	52.98	3.06	9.77	9.20	16.09	2.08	1.84	0.71	4.27	100

The main reason for the occurrence of biotite aggregations was that partial copper-bearing biotite could alter into copper-bearing products through the following two mechanisms, and the copper-bearing products were mainly copper-bearing chlorite, malachite or copper-bearing limonite. Moreover, According to the statistical data of EDS, it was discovered that the average copper contents of chlorite, malachite and limonite in the aggregations were 4.05, 42.21 and 2.94% respectively^8^.

### Alteration mechanism of copper-bearing biotite

It was noticed that a large amount of potassium and silicon were lost in the process of biotite altering into chlorite, and the lost silicon was formed into quartz or amorphous silicon^33^. Ilton and Veblem indicated that the copper content of biotite was related to the supergenesis^22^. As known, the supergene zone can be further divided into the leach cap and the enrichment zone. Leach caps usually have the lowest Cu values, while the enrichment zone has the highest Cu^34^. In this paper, it was discovered that in the enrichment zone, the alteration mechanism of copper-bearing biotite was divided into two types according to the strength of supergenesis. The first was that the copper-bearing biotite altered into copper-bearing chlorite and malachite, and the second was the copper-bearing biotite altering into copper-bearing chlorite and copper-bearing limonite.

#### Copper-bearing biotite → copper-bearing chlorite + malachite

It was proved by Wang *et al*. that in the presence of HCl, biotite could change into chlorite with red Fe_2_O_3_ around its periphery^35^. In this process the unstable FeCl_2_ was released which can self-oxidized to Fe^3+^, and the Fe^3+^ continued to turn to Fe_2_O_3_ under weakly acidic condition. The reaction that biotite changed into chlorite is shown in Eq. (1):

1 $$\mathop{2K(\mathrm{Fe},\,{\mathrm{Mg})}_{3}{{\rm{AlSi}}}_{3}{{\rm{O}}}_{10}{(\mathrm{OH})}_{2}+4{\rm{HCl}}\,\to \,}\limits_{(\mathrm{biotite})}\mathop{{({\rm{Fe}},{\rm{Mg}})}_{5}{{\rm{Al}}}_{2}{{\rm{Si}}}_{3}{{\rm{O}}}_{10}{({\rm{OH}})}_{8}+({\rm{Fe}},\,{\rm{Mg}}){{\rm{Cl}}}_{2}+2{\rm{KCl}}+3{{\rm{SiO}}}_{2}}\limits_{(\mathrm{chlorite})}$$

One can see in Fig. 2 that the area (1) in SEM diagram corresponds to the biotite absorbing a small amount of copper, while area (2) corresponds to the copper-bearing chlorite altered from copper-bearing biotite, and the area (3) is malachite. The specific alteration mechanism of copper-bearing biotite altering to copper-bearing chlorite and carbonate minerals was shown as follows. First, the copper-bearing biotite (EDS1) was transformed from copper-free biotite by releasing out a large amount of iron and magnesium, meanwhile absorbing some copper. Then, in the presence of CO_2_ and H_2_O under natural conditions, the products of copper-bearing chlorite (EDS2) and carbonate were generated, showing in Eq. (2) ^36^. Further, under the severe differentiation condition, more copper which completely replaced magnesium and iron was absorbed by carbonate of (Fe, Mg, Cu)CO_3_, leading to the generation of unstable copper carbonate (CuCO_3_). Thus the stable copper-containing oxide mineral of malachite (EDS3) was created from the unstable copper carbonate (CuCO_3_), the reaction can be seen in Eq. (3).

2 $$\begin{array}{c}\mathop{2{\rm{K}}{({\rm{Fe}},{\rm{Mg}},{\rm{Cu}})}_{3}{{\rm{AlSi}}}_{3}{{\rm{O}}}_{10}{({\rm{OH}})}_{2}}\limits_{({\rm{copper}}-{\rm{bearingbiotite}})}+2{{\rm{CO}}}_{2}\\ +2{{\rm{H}}}_{2}{\rm{O}}\to \mathop{{({\rm{Fe}},{\rm{Mg}},{\rm{Cu}})}_{5}{{\rm{Al}}}_{2}{{\rm{Si}}}_{3}{{\rm{O}}}_{10}{({\rm{OH}})}_{8}}\limits_{({\rm{copper}}-{\rm{bearingchlorite}})}\\ +({\rm{Fe}},{\rm{Mg}},{\rm{Cu}}){{\rm{CO}}}_{3}+{{\rm{K}}}_{2}{{\rm{CO}}}_{3}+3{{\rm{SiO}}}_{2}\end{array}$$

3 $$2{{\rm{CuCO}}}_{3}+{{\rm{H}}}_{2}{\rm{O}}\to {{\rm{Cu}}}_{2}{({\rm{OH}})}_{2}{{\rm{CO}}}_{3}+{{\rm{CO}}}_{2}$$

The ΔrGm values for the Eqs (2) and (3) at 298.15 K are both less than zero, indicating that the processes of reactions (2) and (3) were spontaneous under natural conditions.

#### Copper-bearing biotite → copper-bearing chlorite + copper-bearing limonite

As can be seen in Fig. 3, the area (1) in SEM-EDS represents copper-bearing biotite, area (2) indicates copper-bearing chlorite altered from copper-bearing biotite and area (3) represents copper-bearing limonite with honeycomb voids. According to Eq. (2) above, the alteration mechanism of copper-bearing biotite altering to copper-bearing chlorite and copper-bearing limonite was that, the copper-bearing biotite (EDS1) was transformed from copper-free biotite by releasing out a large amount of iron and magnesium, meanwhile absorbing some copper. In the presence of CO_2_ and H_2_O under natural conditions, the products of copper-bearing chlorite (EDS2) and carbonate were generated. It was discovered that (Fe, Mg, Cu)CO_3_ could continue to be oxidized and hydrolyzed to limonite because of its instability, showing Eq. (4). Besides, owing to the adsorption characteristic^37^, some copper from surrounding can be adsorbed by limonite, resulting in the formation of copper-bearing limonite (EDS3).

4 $$4({\rm{Fe}},{\rm{Mg}},{\rm{Cu}}){{\rm{CO}}}_{3}+3{{\rm{O}}}_{2}+2{{\rm{H}}}_{2}{\rm{O}}\to \mathop{2{{\rm{Fe}}}_{2}{{\rm{O}}}_{3}\cdot {{\rm{H}}}_{2}{\rm{O}}}\limits_{({\rm{copper}}-{\rm{bearing}}\,{\rm{limonite}})}+4({\rm{Mg}},{\rm{Cu}}){{\rm{CO}}}_{3}$$

### Leachable property of copper-bearing minerals

Table 3 shows the L_16_ (4^5^) experimental table of copper leaching rate. The range analysis of copper leaching rate was performed in order to determine the effect of parameters on the leaching, showing in Table 4. It was found that the range values of A, B, C, D and E were 21.526, 11.043, 9.166, 7.586 and 10.610, respectively. Hence, the order of factors influencing the leaching was temperature > sample size > H_2_SO_4_ concentration > leaching time > stirring speed, indicating that temperature has the greatest effect on the leaching.

**Table 3:** L_16_ (4^5^) experimental table of copper leaching rate.

Exp. no.	Experimental factors					Copper leaching rate (%)
	T (°C)	S < 74 μm (%)	t (min)	R (rpm)	${{\bf{C}}}_{{{\bf{H}}}_{2}{\bf{S}}{{\bf{O}}}_{4}}$ (mol/L)	
1	25	40	60	200	0.2	47.694
2	25	60	90	300	0.7	77.748
3	25	80	120	400	1.2	82.033
4	25	100	150	500	1.7	85.930
5	40	40	90	400	1.7	81.462
6	40	60	60	500	1.2	84.098
7	40	80	150	200	0.7	85.586
8	40	100	120	300	0.2	85.119
9	60	40	120	500	0.7	91.369
10	60	60	150	400	0.2	88.881
11	60	80	60	300	1.7	92.781
12	60	100	90	200	1.2	94.488
13	80	40	150	300	1.2	96.024
14	80	60	120	200	1.7	95.793
15	80	80	90	500	0.2	92.508
16	80	100	60	400	0.7	95.182

**Table 4:** Range analysis data of copper leaching rate.

Value name	Mean leaching rate of copper				
	T (°C)	S < 74 μm (%)	t (min)	R (rpm)	C_H2SO4_ (mol/L)
E_1_	73.351	79.137	79.939	80.890	78.551
E_2_	84.066	86.630	86.552	87.918	87.471
E_3_	91.880	88.227	88.578	86.890	89.161
E_4_	94.877	90.180	89.105	88.476	88.992
R	21.526	11.043	9.166	7.586	10.610

**Figure 4:**
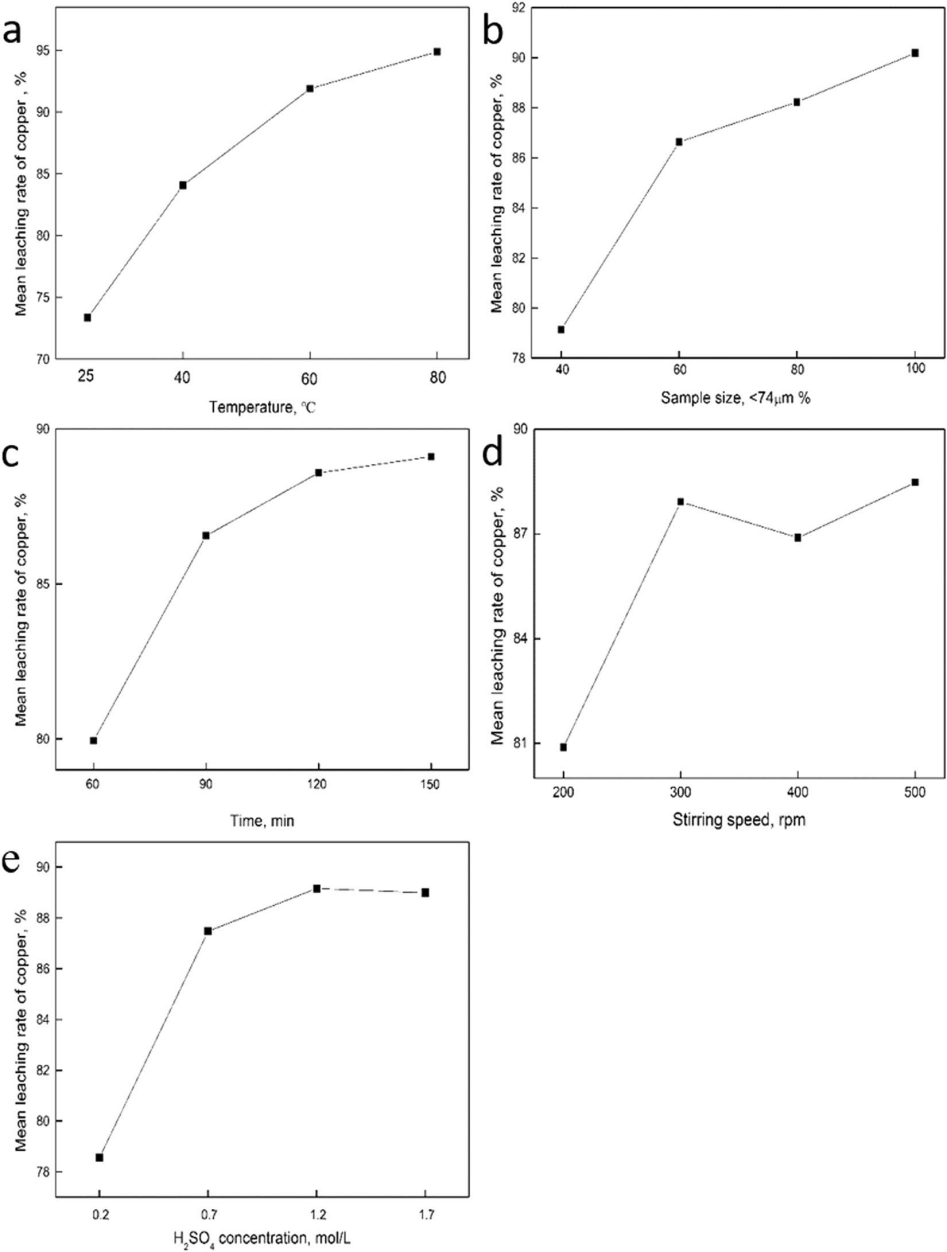
The effect of each factor on mean leaching rate of copper. (**a**) Temperature; (**b**) Sample size; (**c**) Time; (**d**) Stirring speed; (**e**) H_2_SO_4_ concentration.

Figure 4(a–e) represents the effect of each factor on the mean leaching rate of copper. It should be noted that these graphs were only used to show the trend of each factor, rather than predicting other values that were not tested experimentally. From Fig. 4(a), it was seen that as the temperature was increased from 25 to 80 °C, the leaching rate of copper increased remarkably. If the temperature continues to rise, the leaching rate of copper may be better than that at 80 °C. But 80 °C was considered to be the optimum, because of the industrial energy consumption. It was observed from Fig. 4(b) that an increase in particles size <74 μm from 40 to 60% led to the significant increase of copper leaching rate, which due to the increased generation of particle surface area that produced rapider leaching kinetics^4,38^. But when the particle size <74 μm increased from 60 to 100%, the increasing trend decreased. Basing on the grinding energy consumption, the sample particle size <74 μm of 80% was chosen. It was found from Fig. 4(c) that the leaching rate of copper was increased with prolonging time, and 120 min was considered the best. In addition, it was shown from Fig. 4(d) that the leaching rate increased significantly with increasing the stirring speed from 200 to 300 rpm, while which remained almost unchanged as to 500 rpm. Therefore, the optimal stirring speed was considered to be 300 rpm. Moreover, when the H_2_SO_4_ concentration increased from 0.2 to 1.2 mol/L, the leaching rate increased greatly, however, the upward trend decreased from 1.2 to 1.7 mol/L in Fig. 4(e). Thus, considering the corrosion of equipment, 1.2 mol/L was the better option.

**Figure 5:**
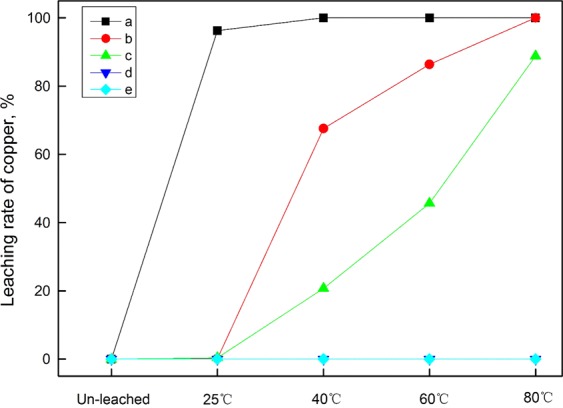
Leaching rate of copper in different occurrence states at different temperatures. (**a**) Copper in mineral state; (**b**) Isomorphic copper; (**c**) Adsorbed copper; (**d**) Copper in cemented body; (**e**) Other bound copper.

To further study the leachable properties of copper-bearing minerals, the leaching experiments were carried out under the optimal conditions at different temperatures. The results indicated that the leaching rates of copper at 25, 40, 60 and 80 °C were 69.35, 82.04, 87.84, and 94.03%, respectively. Figure 5 shows the leaching rate of copper in different occurrence states at different temperatures. It was found from Fig. 5 that at 25 °C the leaching rate of copper in mineral state was 96.21%, while the copper was hardly leached out in other states. With increasing the temperature to 40 °C, the copper in mineral state was entirely leached out. Besides, 67.57 and 20.75% of copper leaching rate in isomorphism and adsorption states were obtained, indicating that isomorphic copper was more easily leached compared with the adsorbed copper at this temperature. When the temperature increased to 60 °C, the leaching rate of copper in adsorption state increased significantly to 45.64%, which was 86.34% of isomorphism. In addition, it was shown that at 80 °C, the isomorphic copper was completely leached, but remaining 11.2% of adsorbed copper un-leached. However, the copper in cemented body of feldspar-quartz-copper-iron and other bound copper were not dissolved throughout the leaching.

**Figure 6:**
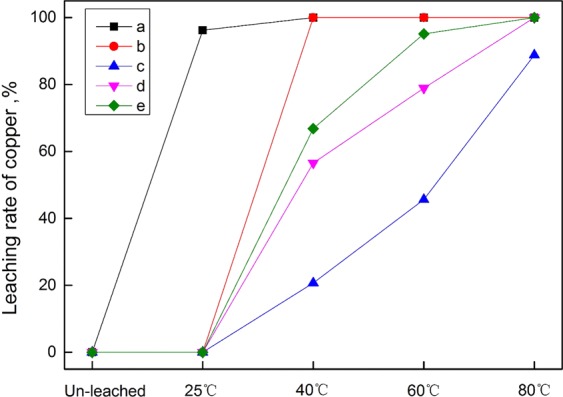
Leaching rate of copper in copper-bearing minerals at different temperatures. (**a**) Malachite, chrysocolla and pseudo-malachite. (**b**) Copper-bearing chlorite. (**c**) Copper-bearing limonite. (**d**) Copper-bearing biotite. (**e**) Copper-bearing muscovite.

**Figure 7:**
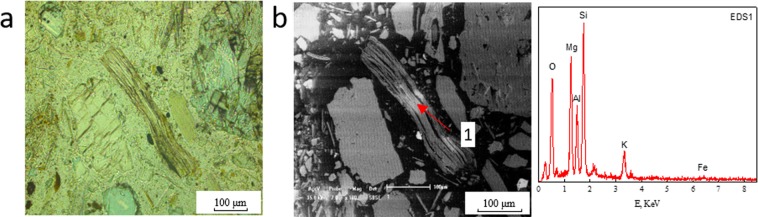
The morphology and elemental characteristics of biotite after leaching at 80 °C. (**a**) Planar polarizing microscopy. (**b**) SEM; EDS1. The spectra corresponds to point 1 in SEM diagram.

Figure 6 shows the leaching rate of copper in copper-bearing minerals at different temperatures. It can be seen that at 25 °C, most of mineral states such as malachite, chrysocolla and pseudo-malachite were leached, while other copper-bearing minerals like biotite, chlorite, muscovite and limonite were hardly leached. With the increase of temperature, the copper in the four minerals was gradually leached out. When the temperature was at 40 °C, 56.52, 66.80 and 20.76% of copper leaching rates for biotite, muscovite and limonite were achieved. Surprisingly, the copper in chlorite was completely leached out, this is due to the fact that in the process of biotite altering into chlorite, the layered structure of chlorite was changed, leading to more copper to be leached out easily^33^. Furthermore, it was found that the copper leaching rate of limonite increased obviously at the range of 40–80 °C. Figure 7 shows the morphology and elemental characteristics of biotite at 80 °C. It was shown from EDS1 that all the copper was dissolved out leaving the unchanged crystal shape of biotite after leaching, indicating that the leachable property of biotite is closely related to its special layered structure^39^. However, 11.2% of copper in limonite was un-leached at 80 °C. In conclusion, the leaching order of copper in different minerals was as follows: malachite, chrysocolla and pseudo-malachite > copper-bearing chlorite > copper-bearing muscovite > copper-bearing biotite > copper-bearing limonite.

## Experimental

### Materials

**Figure 8:**
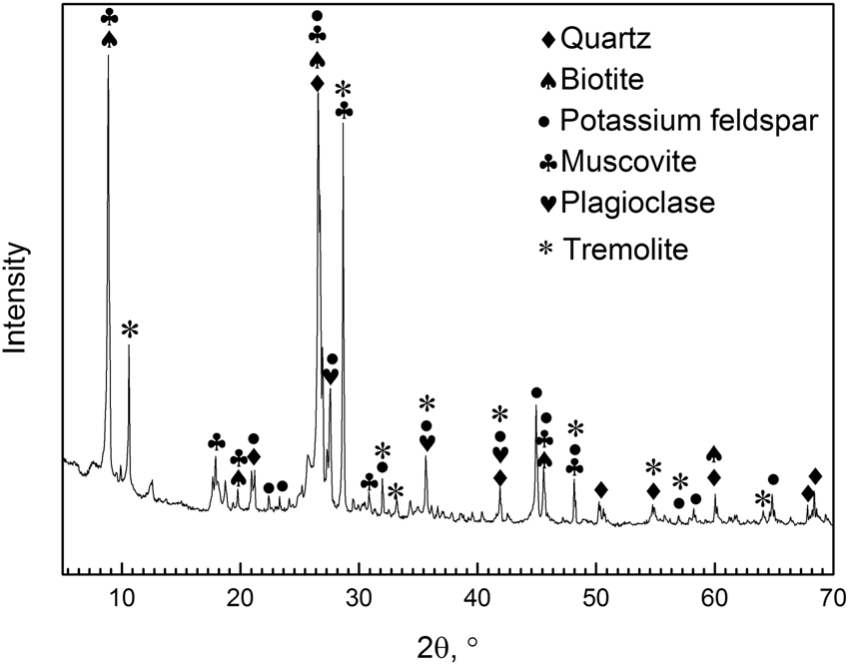
XRD pattern of the copper ores.

The samples used in this study were copper oxide ores obtained by open-pit mining from the Mulyashy Copper Mine in Luanxia, Zambia. The mineralogical process data indicated that there were four occurrence states of copper in copper minerals^8^: copper in mineral state (70%), isomorphic copper (13.32%), adsorbed copper (12.05%) and a few copper in cemented body of feldspar-quartz-copper-iron (2.6%). Other bound copper accounted for 1.53%. The elements of the copper ores were analyzed by XRF (Table 5); Fig. 8 shows the XRD pattern of the copper ores. The particle size distribution of the copper samples was determined by wet sieving (Table 6). Each grain-grade ore was mixed with resins and made into a thin section (4 pieces) to study. The sulfuric acid used in this work was of analytical grade.

**Table 5:** XRF results of the copper ores (Wt%).

SiO_2_	Al_2_O_3_	MgO	K_2_O	Fe_2_O_3_	CaO	CuO	TiO_2_	MnO	BaO
52.9677	15.6791	8.7114	8.6701	6.5438	3.0486	2.6875	0.8048	0.3726	0.1390
P_2_O_5_	Co_2_O_3_	Na_2_O	SO_3_	Cr_2_O_3_	HfO_2_	Rb_2_O	ZrO_2_	NiO	SrO
0.0867	0.0648	0.0533	0.0416	0.0294	0.0231	0.0212	0.0189	0.0153	0.0127

**Table 6:** Particle size distribution of the copper ores (Wt%).

>212 μm	125~212 μm	74~125 μm	<74 μm
30.13	13.80	17.00	39.07

### Characterization

The copper occurrence and alteration mechanism of copper-bearing biotite were studied by analyzing the chemical composition by XRF (ZSX100e), and measuring the phase composition through XRD (PW3040/60) to preliminarily determine the existence of biotite in the ore. The thin sections were examined and analyzed using polarizing microscopy (LEICA-DMLP) and SEM (SHIMADZU SSX-550) equipped with an EDS detector.

### Leaching experiment

The leachable property of copper ores was detected using orthogonal array method. Basing on the previous leaching tests, it was identified that temperature, sample size, leaching time, stirring speed and H_2_SO_4_ concentration could affect the leaching process. Each of five parameters had four levels with an orthogonal array 16 (4^5^)^40^. Low, medium and high levels of the factors are given in Table 7.

**Table 7:** Parameters and levels of the experiments.

Parameters	Experimental levels			
	1	2	3	4
Temperature (°C)	25	40	60	80
Sample size <74 μm (%)	40	60	80	100
Time (min)	60	90	120	150
Stirring speed (rpm)	200	300	400	500
H_2_SO_4_ concentration (mol/L)	0.2	0.7	1.2	1.7

First, the samples were ground in a ball mill to different particle sizes. Raw ores (30 g) of different sizes were added into a 250 mL beaker with 150 mL of sulfuric acid at a certain concentration. The leaching experiment was carried out under water-bath heating at a constant temperature and dynamic stirring. After leaching for a certain time, the solution was filtered, and the filter slag was washed, dried and analyzed by atomic absorption spectroscopy (AAS) to determine the copper content.

## Conclusions

1. There were two kinds of biotite in Mulyashy Copper Mine, Zambia, copper-bearing biotite and copper-free biotite. And the copper–bearing biotite was mainly existed in the form of monomer or aggregations.
2. The alteration mechanism of copper-bearing biotite can be divided into two types according to the strength of epigenesist. First, copper-bearing biotite altered into copper-bearing chlorite and malachite. The second, copper-bearing biotite altered into copper-bearing chlorite and copper-bearing limonite. The leachable property of biotite is closely related to its special structure.
3. The order of factors effecting the copper leaching rate was temperature > sample size > H_2_SO_4_ concentration > leaching time > stirring speed.
4. The leaching order of copper in different minerals at different temperatures was as follows: malachite, chrysocolla and pseudo-malachite > copper-bearing chlorite > copper-bearing muscovite > copper-bearing biotite > copper-bearing limonite.

## Data Availability

All data generated or analysed during this study are included in this published article.
